# A dynamic authorizable ciphertext image retrieval algorithm based on security neural network inference

**DOI:** 10.1371/journal.pone.0309947

**Published:** 2024-10-23

**Authors:** Xin-Yu Zhang, Jing-Wei Hong

**Affiliations:** 1 School of Statistics and Applied Mathematics, Anhui University of Finance and Economics, Beng’ bu, China; 2 College of Computer and Data Science, Fuzhou University, Fuzhou, China; 3 College of Software, Fuzhou University, Fuzhou, China; Jinan University, CHINA

## Abstract

In this paper, we propose a dynamic authorizable ciphertext image retrieval scheme based on secure neural network inference that effectively enhances the security of image retrieval while preserving privacy. To ensure the privacy of the original image and enable feature extraction without decryption operations, we employ a secure neural network for feature extraction during the index construction stage of encrypted images. Additionally, we introduce a dynamic authenticatable ciphertext retrieval algorithm to enhance system flexibility and security by enabling users to quickly and flexibly retrieve authorized images. Experimental results demonstrate that our scheme guarantees data image privacy throughout the entire process from upload to retrieval compared to similar literature schemes. Furthermore, our scheme ensures data availability while maintaining security, allowing users to conveniently perform image retrieval operations. Although overall efficiency may not be optimal according to experimental results, our solution satisfies practical application needs in cloud computing environments by providing an efficient and secure image retrieval solution.

## I. Introduction

With the rapid development of 5G and Internet of Things (IoT) technologies, IoT devices have often uploaded image data to the cloud for storage to prevent image data query attacks. Moreover, while interacting with semi-honest model servers, the analysis of uploaded image data can reduce the risk of servers acquiring sensitive information. However, cloud service providers (CSP) possibly dig and analyze user data without notifying users, which may lead to user privacy leakage. To ensure the data security and the privacy of users, users encrypt the image data before uploading it. Nevertheless, it brings a challenge: the encrypted image data cannot be directly retrieved in its original form. Thus, how to solve the problem of retrieving encrypted image data effectively has become an urgent problem.

Content-based image retrieval (CBIR) is extremely important because of the large amount of sensitive information collected by IoT devices, so secure CBIR of encrypted images on third-party servers or in the cloud, as well as on dedicated private servers, is essential to ensure confidentiality during processing and storage.

Yang et al. [1] proposed a traceable encrypted image retrieval system based on MU-TEIR, which uses convolutional neural networks to extract image feature vectors and construct indexes, encrypts indexes using distributed double-trap public key cryptography system, and encrypts each pixel through standard stream cryptography to protect image content. In addition, the system also uses a watermarking based mechanism to prevent malicious query users from distributing images in a malicious way, and proves through detailed security analysis that it ensures outsourced image and index security as well as query privacy, and can track malicious users. Hazra [2] proposed a design solution for an encrypted image retrieval system to cope with the security problems caused by the exponential growth of image database capacity and wide application. The solution includes encryption algorithm, feature database construction algorithm, image retrieval mechanism, similarity comparison of key features, and supervised learning algorithm to achieve image retrieval. An et al. [3] propose a new method for achieving traceability and encrypted image retrieval in a multi-user environment. Yang et al. [4] used FrSBFMs, a new method based on fractional order weighted spherical Besser-Fourier moments, and QFrSBFMs, developed in combination with quaternion theory, for image analysis, pattern recognition and computer vision. Experimental results show that these methods are effective in image reconstruction capability, pattern recognition accuracy and zero-watermark verification.

Recently, a number of scientists have developed several SSE algorithms to provide ciphertext search on encrypted cloud data. Multi-keyword search is one of the fundamental strategies [5–9]. Compared to single-keyword search [10–13], multi-keyword search can be effective in improving the effectiveness of search results [14–16], In addition, the literatures [6, 17, 18] have further extended the application scope of multi-keyword search by exploring the search effect and performance in different scenes. Upon this foundation, a number of researchers [19–21] have begun to make further discussion and experimental validation for exact keyword search. They significantly have improved the efficiency and accuracy of exact keyword search by improving the search algorithms and optimizing the index structure. However, these programs only support exact keyword searches, considering that users may forget or enter the wrong keywords during a search, they proposed fuzzy search. Li et al. have proposed an efficient and Verifiable Ranked Fuzzy Multi-keyword (VRFMS) Search scheme [22], its scheme uses locality-sensitive hashing and bloom filter to implement fuzzy keyword search. It also employs Term Frequency-Inverse Document Frequency (TF-IDF) to sort the relevant results, significantly enhancing the search accuracy. Then, Chen et al. have proposed an Efficient Leakage-resilient Multi-keyword Fuzzy Search (ELiMFS) [23] framework over encrypted cloud data. In this framework, a novel two-stage index structure is exploited to ensure that search time is independent of file set size, it proved to be efficient and leakage-resilient. However, cloud severs are semi-honest model, which is not always true in actual situation. These schemes for multi-keyword searches have not analyzed the security of their retrieval keywords.

Lately, researchers have actively integrated the concepts of homomorphic encryption and federal learning into the design of ciphertext retrieval schemes, introducing not only a series of innovative homomorphic encryption methods [24–27], but also numerous ciphertext retrieval solutions for the field of data security and privacy protection [27–29]. Having accomplished this, Arazzi et al. [30] have further developed an innovative co-learning scheme that cleverly utilizes homomorphic encryption to safeguard the privacy of users’ personal data in IoT scenes, successfully achieving the objective of enhancing privacy protection while maintaining data retrieval efficiency.

Moreover, some innovative schemes [31–33] have now provided users with secure and efficient access to information, minimizing the risk of unauthorized information leakage while guaranteeing accurate information retrieval. Notably, Meftah et al. [34] have made a significant breakthrough in the evaluation of neuron activation functions, developing a low-depth, batched neuron scheme that directly evaluates multiple quantized ReLU-activated neurons on encrypted data, eliminating the need for complex approximate simulation processes. This seminal innovation not only enhances the overall security of the retrieval process but also significantly reduces the risk of unauthorized leakage of users’ information. However, these schemes have to perform a considerable number of bootstrap operations, thereby increasing the complexity of the computation, potentially leading to latency of the search, as well as affecting the security.

In recent years, IoT security has attracted much attention. Dynamic Searchable Symmetric Encryption (DSSE) [35–38] schemes have made significant progress in achieving efficient search while protecting the privacy of data. Stefanov et al. have proposed an effective DSSE scheme [39]. In addition, Wang et al. have proposed an efficient multiuser forward privacy searchable encryption scheme with dynamic certification [40], though these schemes do not ensure the backward privacy, which restricts their applications to some extent. On the basis of this, Li et al. further have presented an efficient dynamic searchable symmetric encryption scheme for healthcare cloud data security [41], which has combined *k*-nearest neighbor (KNN) and attribute-based encryption (ABE) techniques to achieve the forward and backward privacy protection. Moreover, in order to solve the real-time renewal problem, researchers have successively proposed schemes [42–44]. It is notable that, aiming to support a wider range of practical application scenarios, Ma et al. have proposed a DSSE scheme that supports recoverable keyword masking in a multiuser environment [45], which has used trapdoor substitutions and encryption to achieve both the forward and backward security. Nevertheless, the indexes of these schemes have been built locally, once they have been compromised, it may be possible for an attacker to infer the contents of the encrypted documents from the indexes. In addition, local index updating has to be done manually and is not available in real-time, which may lead to the inaccuracy of search results.

Therefore, we propose a novel searchable encryption scheme based on security neural network to support multi-keyword dynamic authorization based on the above work. The solution supports multi-keyword search while encrypting and protecting the data and computation process during the search process, which effectively improves data security and privacy protection in the IoT environment. It is described as follows:

1. We use homomorphic neural network inference to build the ciphertext index, which can realize the indexing of ciphertexts without decryption, effectively avoiding the privacy leakage problem that may be triggered by the index building process. In addition, homomorphic neural network inference has better fault tolerance and robustness, which can improve the accuracy and reliability of ciphertext indexing.
2. We propose a ciphertext indexing retrieval scheme with dynamic authorization, which can dynamically generate authorization keys according to the user’s query requirements and achieve support for multi-keyword search.
3. We adopt an optimization algorithm to optimize the retrieval process, which improves the retrieval efficiency and accuracy. Through the combination of dynamic authorization and cryptographic protection, we successfully achieve the goal of multi-keyword search with privacy protection.

The rest of the paper is organized as follows: we introduce the required math relevant knowledge as well as cryptography related knowledge. It includes mainly bilinear pairs, hard problems in section II. Then, in section III, we provide the formal definition of the scheme as well as give the specific construction of the scheme. In section IV, we further carry out the security proof and correctness analysis of the constructed scheme, then we analyze the performance of the construction scheme and compare it with similar schemes. Finally, we make conclusions in Section V.

## II. Difficult issues

Define 1: Given that *p* is a large prime, *G*_1_×*G*_2_ is a cyclic group of order *p*, and g is a generating element of group *G*_1._ Define a bilinear mapping *e*:*G*_1_×*G*_2_→*G*_2_ having properties (1)–(3):

1. Bilinear: ∀*g*∈*G*_1_, $a,b\in Z_{p}^{*}$, with $e(g^{a},g^{b})=e{\left(g,g\right)}^{ab}$.
2. Non-degeneracy: ∃*g*∈*G*_1_, making *e*(*g*,*g*)≠1.
3. Computability: ∀*g*∈*G*_1_, there exists an efficient algorithm to compute *e*(*g*,*g*).

Define 2: Choose $a,b\in Z_{p}^{*}$ randomly, and provide a tuple (*P*,*aP*,*bP*). Then calculate *abP* is hard. Define *negl*(*λ*) is a negligible advantage, assuming that the adversary *A* computes *abP* in polynomial time with an advantage of $Adv_{A}^{CDH}(\lambda )$, then we compute: $Adv_{A}^{CDH}(\lambda )\leq negl(\lambda )$.

Define 3: Given a random selection of $a,b,c\in Z_{p}^{*}$, and a tuple (*g*,*g*^*a*^,*g*^*b*^,*g*^*c*^)∈*G*_1_, computing *e*(*g*,*g*)^*abc*^ is hard.

Define 4: Given an array $\left(g_{1},ag_{1},bg_{1},cg_{1},e{\left(g_{1},g_{1}\right)}^{abc}\right)$ and an array (*g*_1_,*ag*_1_,*bg*_1_,*cg*_1_,*R*), where $a,b,c\in Z_{p}^{*}$ and *R*∈*G*_2_, distinguishing between *e*(*g*_1_,*g*_1_)^*abc*^ and *R* is difficult. Additionally, *negl*(*λ*) is a negligible advantage, assuming that the advantage of attacker *A* in distinguishing *e*(*g*_1_,*g*_1_)^*abc*^ and *R* in polynomial time is $Adv_{A}^{DBDH}(\lambda )$. Then, we compute $Adv_{A}^{DBDH}(\lambda )\leq negl(\lambda )$.

Define 5: Given a tuple (*P*,*aP*,*bP*,*cP*). Besides it is difficult to determine *c* = *ab*(mod *q*). Define *negl*(*λ*) is a negligible advantage. If adversary *A* has an advantage of $Adv_{A}^{DDH}(\lambda )$ in solving the DDH tough problem in polynomial time, then we compute: $Adv_{A}^{DDH}(\lambda )\leq \mathrm{negl}(\lambda )$.

As a result, in this work, we will concentrate on the difficult problems from Definition 1 through Definition 5. We will present a dynamic authorizable ciphertext picture retrieval scheme based on security neural network inference. The scheme aims to achieve secure and efficient retrieval of image information by combining advanced neural network and encryption methods, while also ensuring that in a dynamic environment, users can perform flexible and controllable authorization operations based on their own needs.

## III. Program description

In this paper, we choose to use the homomorphic neural network proposed by Microsoft to extract ciphertext image features directly on CSP. For details, please refer to [46].

The system model of the scheme in this chapter contains a total of five entities, such as IoT user *U*, cloud computing service provider *CSP*_*C*_, cloud storage service provider *CSP*_*S*_, trusted authorization *TA* and query user *QU*.

1. Trusted authorization *TA*: run the system parameters, generate public-private key pair of *U* and *QU*, distribute the key pair to *U* and *QU*, and send authorization pair to cloud computing service provider *CSP*_*C*_.
2. IoT user *U*: provide symmetric encrypted data to *CSP*_*S*_, homomorphic encrypted data to *CSP*_*C*_, and send public-private key pair request to *TA*.
3. Cloud storage service provider *CSP*_*S*_: accept and store the IoT user’s symmetric encrypted data as well as the ciphertext of the file identifier encrypted by *CSP*_*C*_, then deliver the file identifier data pair to *QU*.
4. Query user *QU*: send a retrieval request to cloud computing service provider *CSP*_*S*._ Receive the authorized ciphertext from *CSP*_*C*_, decode it with the private key, and then upload the keyword trapdoor to the storage service provider *CSP*_*C*_, for searching. Receive and decrypt the encrypted search results returned by *CSP*_*S*_ for plaintext data. Obtain and decrypt the corresponding data files.
5. Cloud computing service provider *CSP*_*C*_: Receive the matching authorization pair from *TA*, search keywords by *QU*, and homomorphic encrypted data sent by IoT user *U*. It also conducts a computational task to send the file identifier ciphertext to *CSP*_*S*_. Meanwhile, *CSP*_*c*_ will perform feature extraction on homomorphic encrypted images.

Based on the above description, the program of this chapter consists of the following seven algorithms:

1. *Setup*(*β*)→*paras*: *TA* inputs security parameters *β*, outputs public system parameters *pub*_*params*, private system parameters *priv*_*params*.
2. $KeyGen(pub\_params)\to ((pk_{qu},s_{qu}),(pk_{u},s_{u}))$: *TA* inserts system parameters, generates public-private key pair for retrieve user *QU* and user *U*.
3. $PairGen(ind(w),pk_{qu})\to (pk_{A},s_{A})$: *TA* generates matching authorization pair (*pk*_*A*_,*s*_*A*_) and sends to cloud service provider *CSP*_*C*_.
4. $SymEnc(data,\;s_{u})\to C_{s}$: User *U* uses private key *s*_*u*_ to symmetrically encrypt original data, generates ciphertext *C*_*s*_ and sends it to cloud storage service provider *CSP*_*S*_.
5. $HomEnc(data,\;pk_{u})\to C_{H}$: User *U* uses public key *pk*_*u*_ to homomorphically encrypt original data, generates ciphertext *C*_*H*_ and sends it to cloud computing service provider *CSP*_*C*._
6. $Update(pub\_params,priv\_params,pk_{qu},pk_{u},pk_{A},s_{A},ind(w),W)\to C$: *CSP*_*C*_ inputs the public system parameter *pub*_*params*, the private system parameter *priv*_*params*, the public key *pk*_*qu*_ of *QU* and the public key *pk*_*u*_ of user *U*. Additionally, *CSP*_*C*_ matches authorization pair (*pk*_*A*_,*s*_*A*_), set of file identifiers *ind*(*w*) and set of keywords *W*. Finally, *CSP*_*C*_ outputs keyword ciphertext, file identifier ciphertext and authorization ciphertext.
7. $Trapdoor(pub\_params,priv\_params,s_{qu},w_{i})\to T_{w_{i}}$: *QU* executes this algorithm, inputs the public system parameter *pub*_*params*, private system parameter *priv*_*params*, its own private key *s*_*qu*_ and keyword *w*_*i*_. *QU* outputs the keyword search trapdoor $T_{w_{i}}$.
8. $Search(pub\_params,priv\_params,C,T_{w_{i}})\to (cert_{w_{i}}\|ind_{w_{i}})$: *QU* inputs public system parameter *pub*_*params*,, private system parameter *priv*_*params*, ciphertext *C*, keyword search trap $T_{w_{i}}$, and outputs data pair $cert_{w_{i}}\|ind_{w_{i}}$.
9. $Decrypt(pub\_params,priv\_params,CE_{w_{ij}}(cert_{w_{i}}\|ind_{w_{i}}))\to ind_{w_{i}}$: *QU* executes this algorithm, inputs system parameters, authorization ciphertext, data pair $cert_{w_{i}}\|ind_{w_{i}}$, and outputs file identifier $ind_{w_{i}}$.

(1) System establishment algorithm: *Setup*(*β*)→*paras*

*TA* executes this algorithm, inputs the system security parameters *β*, establishes two cyclic groups *G*_*1*_ and *G*_*2*_ of order p in the system to define the bilinear mapping *e*:*G*_1_×*G*_1_→*G*_2_, selects the generating element *M* of the cyclic group *G* to generate the set of file identifiers *ind*(*w*) and the set of keywords *C*, and |*ind*(*w*)| denotes the binary length of the set of file identifiers *ind*(*w*). Define three secure hash functions:

$$H_{0}:{\left\{0,1\right\}}^{*}\in G_{1},\;H_{1}:G_{2}\in {\left\{0,1\right\}}^{\beta },\;H_{2}:G_{2}\in {\left\{0,1\right\}}^{1+\beta +|ind(w)|}$$ (1)

Finally, *TA* outputs the public system parameters

$$pub\_params=(\beta ,p,G_{1},G_{2},M,e,H_{0},H_{1},H_{2},ind(w),W)$$ (2)

Meanwhile, *TA* keeps the undisclosed system parameters: *priv*_*params* = (*r*_*f*_,*r*_*s*_,*r*_*k*_). *r*_*f*_ is the random number used in file identifier generation, *r*_*s*_ is the random number used in key generation, and *r*_*k*_ is the random number used in search operation.

(2) Key generation algorithm: $KeyGen(pub\_params)\to ((pk_{qu},s_{qu}),(pk_{u},s_{u}))$

*TA* inputs system parameter *pub*_*params*, randomly select $z_{1},z_{2}\in Z_{p}^{*}$, and lets *s*_*qu*_ = *z*_1_, *s*_*u*_ = *z*_2_. Additionally, *TA* calculates $pk_{qu}=M^{z_{1}}$, $pk_{u}=M^{z_{2}}$ and uses *(pk*_*qu*_,*s*_*qu*_), (*pk*_*u*_,*s*_*u*_) as the public and private keys.

$PairGen(ind(w),pk_{ru})\to (pk_{A},s_{A})$: *TA* generates a matching authorization pair (*pk*_*A*_,*s*_*A*_) and sends it to the cloud computing service provider *CSP*_*C*_.

$SymEnc(data,\;s_{u})\to C_{s}$: User *U* uses private key *s*_*u*_ to symmetrically encrypt original data, generates ciphertext *C*_*s*_ and sends it to cloud storage service provider *CSP*_*S*_.

$HomEnc(data,\;pk_{u})\to C_{H}$: User *U* uses public key *pk*_*u*_ to homomorphically encrypt original data, generates ciphertext *C*_*H*_ and sends it to cloud computing service provider *CSP*_*C*_.

(3) Data update: $Update(pub\_params,priv\_params,pk_{qu},pk_{u},pk_{A},s_{A},ind(w),W)\to C$

*CSP*_*C*_ inputs public system parameter *pub*_*params*, private system parameter *priv*_*params*. Subsequently, *CSP*_*C*_ retrieves user’s public key *pk*_*qu*,_ user’s public key *pk*_*u*_, and keyword set *W* where $W=\{w_{1},w_{2}\cdots w_{i}\cdots w_{n}\}$. The set of file identifiers *ind*(*w*), where $ind(w)=\{ind_{w_{1}},ind_{w_{2}},ind_{w_{3}},...,ind_{w_{n}}\}$, matching authorization pair (*pk*_*A*_,*s*_*A*_).

*CSP*_*C*_ performs the following operations to generate the ciphertext.

(1) Upload keyword ciphertext generation algorithm

*CSP*_*C*_ retrieves whether exists, where *OC* is the set of keyword ciphertexts for *w*_*i*_, If (*w*_*i*_,*OC*) = ⊥, it computes $OC_{w_{i}}=H_{1}(e(pk_{u},H_{0}{\left(w_{i}\right)}^{s_{0}}))$.

(2) Retrieve the keyword ciphertext generation algorithm

*QU* retrieves whether (*w*_*i*_,*R*) exists, where *R* is the set of keyword ciphertexts for *w*_*i*_. If (*w*_*i*_,*R*) = ⊥, it calculates $R_{w_{i}}=H_{1}(e(pk_{qu},H_{0}{\left(w_{i}\right)}^{s_{0}}))$. Where *s*_*0*_ is a random number that needs to be negotiated in advance between the user and the cloud storage service center, then the keyword ciphertext $OC_{w_{i}}$ is stored into the keyword collection *OC* and updates the *OC*.

(3) Authorization ciphertext generation algorithm

Randomly select $a\in Z_{p}^{*}$, $cert_{w_{i}}\in {\left(0,1\right)}^{\lambda }$, compute the hash value of retrieved user ID *h*_*ID*_ = *H*_0_(*ID*).Subsequently, obtain the counter value *C* = *counter*(*ID*),and calculate the hash value of the counter *h*_*C*_ = *H*_0_(*C*). Additionally, select a random number $r\in Z_{p}^{*}$ and calculate the random factor *random* = *H*_0_(*r*) to compute the authorization ciphertext:

$$d_{auth}=H_{0}(ID)+H_{0}(r/a+H_{0}(C))$$ (3)

$$CE_{w_{i1}}=H_{2}(e(pk_{qu},H_{0}{\left(w_{i}\right)}^{d_{\mathrm{auth}}}))⊕{\mathrm{cert}}_{w_{i}}$$ (4)

$$CE_{w_{i2}}=M^{d_{\mathrm{auth}}}$$ (5)

Let $CE_{w_{i}}=(CE_{w_{i1}},CE_{w_{i2}})$ be the authorization ciphertext of *QU*, where $cert_{w_{i}}$ is the authorization certificate.

(3) File identifier ciphertext generation algorithm

Select the random number $b\in Z_{p}^{*}$, $cert_{w_{i}}\in {\left(0,1\right)}^{\lambda }$, calculate the hash value of user ID *h*_*ID*_ = *H*_0_(*ID*), get the counter value *C* = *counter*(*ID*), and calculate the hash value of counter *h*_*C*_ = *H*_0_(*C*). Additionally, select the random number $r\in Z_{p}^{*}$, calculate the random factor *random* = *H*_0_(*r*) and the hash value *h*_*ind*_ = *H*_0_(*w*_*i*_) of the file identifier. Finally, calculate:

$$d_{cind}=H_{0}(ID)+H_{0}(r/b+H_{0}(C))$$ (6)

$$IC_{w_{i}}=H_{2}(e(pk_{u},H_{0}{\left(w_{i}\right)}^{b}))⊕(cert_{w_{i}}\|ind_{w_{i}}\|OC_{w_{i}}\|h_{ID})$$ (7)

$$I_{bw_{i}}=M^{b}$$ (8)

Then $\left(IC_{w_{i}},I_{bw_{i}}\right)$ is the file identifier ciphertext for identifier ciphertext $ind_{w_{i}}$.

(4) *CSP*_*C*_ stores $\left(IC_{w_{i}},I_{bw_{i}}\right)$ into the ciphertext collection *C* and updates the ciphertext collection. Subsequently *CSP*_*C*_ uploads *C* into *CSP*_*S*_ and sends the authorization ciphertext $CE_{w_{i}}$ to *QU* over a secure channel.
(4) Trap generation algorithm: $Trapdoor(pub\_params,priv\_params,s_{qu},w_{i})\to T_{w_{i}}$

*QU* inputs the public system parameter *pub*_*params*, the private system parameter *priv*_*params*, its own private key *s*_*qu*_ and the keyword *w*_*i*_. Additionally, *h* is the hash function and it outputs the keyword search trapdoor computation $T_{w_{i}}=H_{0}{\left(h(w_{i})+h(ID)\right)}^{s_{ru}}$.

(5) Search Algorithm: $Search(pub\_params,priv\_params,C,T_{w_{i}})\to (cert_{w_{i}}\|ind_{w_{i}})$

After receiving the uploaded keyword trap by *QU*, *CSP*_*C*_ performs the following operations:

Obtain the keyword ciphertext $OC_{w_{i}}$ by computation

$$\begin{array}{l} Pairing=H_{1}(e(pk_{A},T_{w_{i}}))\\ \;=H_{1}(e(M^{s_{A}},h(w_{i})+h(ID)))\\ \;=H_{1}(e(pk_{qu},h(\mathrm{value})+s_{A}))\\ \;=OC_{w_{i}} \end{array}$$

$$\begin{array}{l} Pairing=H_{1}(e(pk_{A},T_{w_{i}}))\\ \;=H_{1}(e(M^{s_{A}},h(w_{i})+h(ID)))\\ \;=H_{1}(e(pk_{qu},h(value)+s_{A}))\\ \;=OC_{w_{i}} \end{array}$$

Retrieve the keyword ciphertext *C* from the ciphertext set $\left(IC_{w_{i}},I_{bw_{i}}\right)$, and compute $(cert_{w_{i}}\|{\mathrm{ind}}_{w_{i}}\|OC_{w_{i}})=H_{2}(e(I_{bw_{i}},T_{w_{i}}))⊕IC_{w_{i}}$.

After obtaining the data pair $\left(cert_{w_{i}}\|{\mathrm{ind}}_{w_{i}}\|OC_{w_{i}}\right)$ using the previously mentioned calculation, *CSP*_*S*_ provides the data pair $\left(cert_{w_{i}}\|{\mathrm{ind}}_{w_{i}}\|OC_{w_{i}}\right)$ back to *QU*.

(6) Decryption algorithm $Decrypt(pub\_params,priv\_params,CE_{w_{ij}}(cert_{w_{i}}\|ind_{w_{i}}))\to ind_{w_{i}}$

When *QU* receives the data pair *CSP*_*S*_ returned from $\left(cert_{w_{i}}\|ind_{w_{i}}\right)$, *QU* computes $\left(cert_{w_{i}}\|pk_{u})=CE_{w_{i1}}⊕H_{2}(e(CE_{w_{i2}},H_{0}{\left(w_{i}\right)}^{s_{qu}})\right)$.

Upon obtaining the data pair $\left(cert_{w_{i}}\|pk_{u}\right)$, *QU* can access the corresponding data file through the file identifier since *pk*_*u*_ is known and can thus retrieve the authorization certificate $cert_{w_{i}}$ and file identifier $ind_{w_{i}}$.

## IV. Experimental demonstration and proof of safety

### 1. Proof of security

Theorem 1: Define the hash function *H*_*0*_, *H*_*1*_, *H*_*2*_ are collision-safe hash functions, and homomorphic encryption relies on the fact that the RLWE problem has no interaction process. As a result, the encryption process is secure, and its proof can be disregarded in this paper. Then the CBDH hard problem is established. The dynamic searchable encryption scheme proposed in this paper satisfies the semantic security.

Proof: If there exists an adversary *A* who can crack the scheme of this chapter in polynomial time by a non-negligible margin, then there exists a challenger *B* who can crack the CBDH hard problem in polynomial time by a non-negligible margin. Challenger *B* and adversary *A* have the following question and answer session.

*Setup*: Challenger *B* inputs the system security parameters *λ*, and runs the system building algorithm to generate the system public parameters.


$$pub\_params=(\beta ,p,G_{1},G_{2},M,e,H_{0},H_{1},H_{2},ind(w),W)$$ (9)

$$priv\_params=(r_{f},r_{s},r_{k})$$ (10)

The public parameter is returned to adversary *A* and the private parameter is not returned to *A*.

*KeyGen*: The challenger *B* runs the key generation algorithm to generate the public-private key pair of *QU* and *U*, and sends the public key pair of *QU* and *U* to the adversary.

Phase 1: Adversary *A* makes the following queries adaptively in polynomial time.

(1) *H*_0_ query: Challenger maintains *H*_*0*_ -list $L_{H_{0}}=\emptyset \subseteq G_{1}\times Z_{p}^{*}\times {\left\{0,1\right\}}^{*}$, inputs keyword *w*, chooses $x\in Z_{p}^{*}$ at random, computes *z* = *M*^*x*^, inserts tuple (*w*,*x*,*z* = *M*^*x*^) into list $L_{H_{0}}$ and outputs *z*.
(2) *H*_1_ query: The challenger maintains *H*_*1*_ list $L_{H_{1}}=\emptyset \subseteq G_{1}\times {\left\{0,1\right\}}^{\beta }$, computes *j*∈*G*_2_ as input, chooses $y\in {\left\{0,1\right\}}^{1+\beta +|ind(w)|}$ at random, inserts the tuple (*v*,*y*) into the list $L_{H_{2}}$ and outputs *y*.
(3) *H*_*2*_ query: The challenger maintains *H*_*2*_ list $L_{H_{2}}=\emptyset \subseteq G_{1}\times {\left\{0,1\right\}}^{1+\beta +|ind(w)|}$, randomly chooses *j*∈*G*_2_ as input, randomly selects $y\in {\left\{0,1\right\}}^{1+\beta +|ind(w)|}$, inserts tuple (*v*,*y*) into list $L_{H_{2}}$ and outputs *y*.
(4) Update query: Adversary *A* sends an update query to challenger *B* with keyword *w*∈*W* and file identifier *ind*_*w*_∈*ind*(*w*) The challenger takes the subsequent steps:

(1) The challenger retrieves whether (*w*,*OC*) exists, and if (*w*,*OC*) = ⊥, computes $OC_{w}=H_{1}(e(pk_{qu},H_{0}{\left(w_{i}\right)}^{s_{0}}))$, where *OC* is the set of keyword ciphertexts of *w*_*i*_. The challenger stores the keyword *OC*_*w*_ into *OC* and updates it.
(2) The challenger randomly selects $a\in Z_{p}^{*}$, $cert_{w}\in {\left(0,1\right)}^{\lambda }$ and calculates the hash value *h*_*ID*_ = *H*_0_(*ID*) of the retrieved user ID. After that it obtains the counter value *C* = *counter*(*ID*) and computes the hash value *h*_*C*_ = *H*_0_(*C*) of the counter. Then, it chooses random number $r\in Z_{p}^{*}$, computes the random factor *random* = *H*_0_(*r*). Finally, it calculates the authorization ciphertext:

$$d_{auth}=H_{0}(ID)+H_{0}(r/a+H_{0}(C))$$ (11)

$$CE_{w_{1}}=H_{2}(e(pk_{qu},H_{0}{\left(w\right)}^{d_{auth}}))⊕cert_{w}$$ (12)

$$CE_{w_{2}}=M^{d_{auth}}$$ (13)

*cert*_*w*_ is the authorization certificate.

The challenger randomly picks $b\in Z_{p}^{*}$ to select the random number $b\in Z_{p}^{*}$, *cert*_*w*_∈(0,1), calculates the hash value of the user ID *h*_*ID*_ = *H*_0_(*ID*), obtains the value of the counter *C* = *counter*(*ID*) and computes the hash value of the counter *h*_*C*_ = *H*_0_(*C*). The challenger chooses random number $r\in Z_{p}^{*}$, calculates the random factor *random* = *H*_0_(*r*) and the hash value *h*_*ind*_ = *H*_0_(*w*_*i*_) of the file identifier. Then, it calculates the file identifier ciphertext:

$$d_{cind}=H_{0}(ID)+H_{0}(r/b+H_{0}(C))$$ (14)

$$IC_{w}=H_{2}(e(pk_{u},H_{0}{\left(w\right)}^{b}))⊕(cert_{w}\|ind_{w}\|OC_{w}\|h_{ID})$$ (15)

$$I_{bw}=M^{b}$$ (16)

(4) The challenger stores (*IC*_*w*_,*I*_*bw*_) in the ciphertext set *C*, and updates the ciphertext set.
(5) Trap query: Adversary *A* chooses a keyword w, and sends a trap query to the challenger. After that, the challenger computes $T_{w}=H_{0}{\left(h(w)+h(ID)\right)}^{s_{qu}}$ and sends it to the adversary *A*.
(6) Search query: Adversary *A* selects a search trap *T*_*w*_ and sends a search query to the challenger. The challenger executes the search algorithm and returns the result to adversary *A*.

Challenge phase: After phase 1, the adversary sends two challenge pairs (*w*_0_,*ind*(*w*_0_)), (*w*_1_,*ind*(*w*_1_)) to challenger *B*. The challenger randomly selects and executes the update algorithm. The challenger randomly chooses *b*∈{0,1} and uses an updating method to construct a ciphertext, which is then returned to adversary *A*.

Phase 2: The adversary can continue to send update queries, trapdoor queries, and search queries to the challenger, but it cannot continue to use the keywords *w*_0_ and *w*_1_ for queries.

Guessing phase: The opponent outputs *b*′∈{0,1}, and if *b*′ = *b*, the adversary wins the game. Otherwise, the adversary loses.


$$Adv_{A}^{CBDH}(\beta )=\mathrm{Pr}[b=b^{\prime }]‐\frac{1}{2}$$ (17)

However, since the CBDH problem is hard, the probability $Adv_{A}^{CBDH}(\beta )$ that the adversary wins the game in polynomial time is negligible, hence the dynamically searchable encryption scheme satisfies semantic security.

### 2. Correctness analysis

The correctness of the scheme proposed in this chapter in terms of whether the *QU* can correctly obtain the document identifier $ind_{w_{i}}$ from the *CSP*_*C*_ is analyzed in two main ways:

Based on the keyword trap a submitted by *QU* and the public key *pk*_*qu*_ of *QU*, *CSP*_*C*_ can return $ind_{w_{i}}$ to *QU* by calculation.

Given a keyword trap $T_{w_{i}}$, *CSP*_*C*_ can return all file identifiers c containing the keyword *w*_*i*_ by computation.

First, analyze requirement (1): after receiving the keyword trapdoor submitted by *QU*, *CSP*_*C*_ obtains the keyword ciphertext $OC_{w_{i}}$ by calculating $R_{w_{i}}=H_{1}(e(pk_{qu},H_{0}{\left(w_{i}\right)}^{s_{0}}))$; after that, it retrieves $\left(IC_{w_{i}},I_{bw_{i}}\right)$ from the ciphertext collection *C* and further calculates:

$$(cert_{w_{i}}\|ind_{w_{i}}\|OC_{w_{i}})=H_{2}(e(I_{bw_{i}},T_{w_{i}}))⊕IC_{w_{i}}$$ (18)

*CSP*_*S*_ obtain the file identifier data pair $\left(cert_{w_{i}}\|ind_{w_{i}}\right)$ and send it to *QU*, *QU* calculates after receiving the data pair sent by *CSP*_*S*_:

$$\left(cert_{w_{i}}\|pk_{u})=CE_{w_{i1}}⊕H_{2}(e(CE_{w_{2}},H_{0}{\left(w_{i}\right)}^{s_{qu}})\right)$$ (19)

Obtain the data pair $\left(cert_{w_{i}}\|pk_{qu}\right)$, and because *pk*_*u*_ is known, the authorization certificate $cert_{w_{i}}$ can be obtained, and then the file identifier $ind_{w_{i}}$, where $\left(CE_{w_{i_{1}}},CE_{w_{i2}}\right)$ is the authorization ciphertext sent by *CSP*_*C*_ to *QU*. From the above process, it can be found that *CSP*_*C*_ needs to obtain the search trap $T_{w_{i}}$ submitted by *QU* if it wants to compute the keyword ciphertext $OC_{w_{i}}$ and the data pair $\left(cert_{w_{i}}\|pk_{qu}\right)$. The search trap is generated by constructing the private key of *QU*, and based on the unidirectionality of the hash function and the computational difficulty of the CBDH problem, the adversary is unable to forge the private key of *QU* by computation, and thus is unable to forge the keyword trap.

### 3. Experimental demonstration

**Fig 1:**
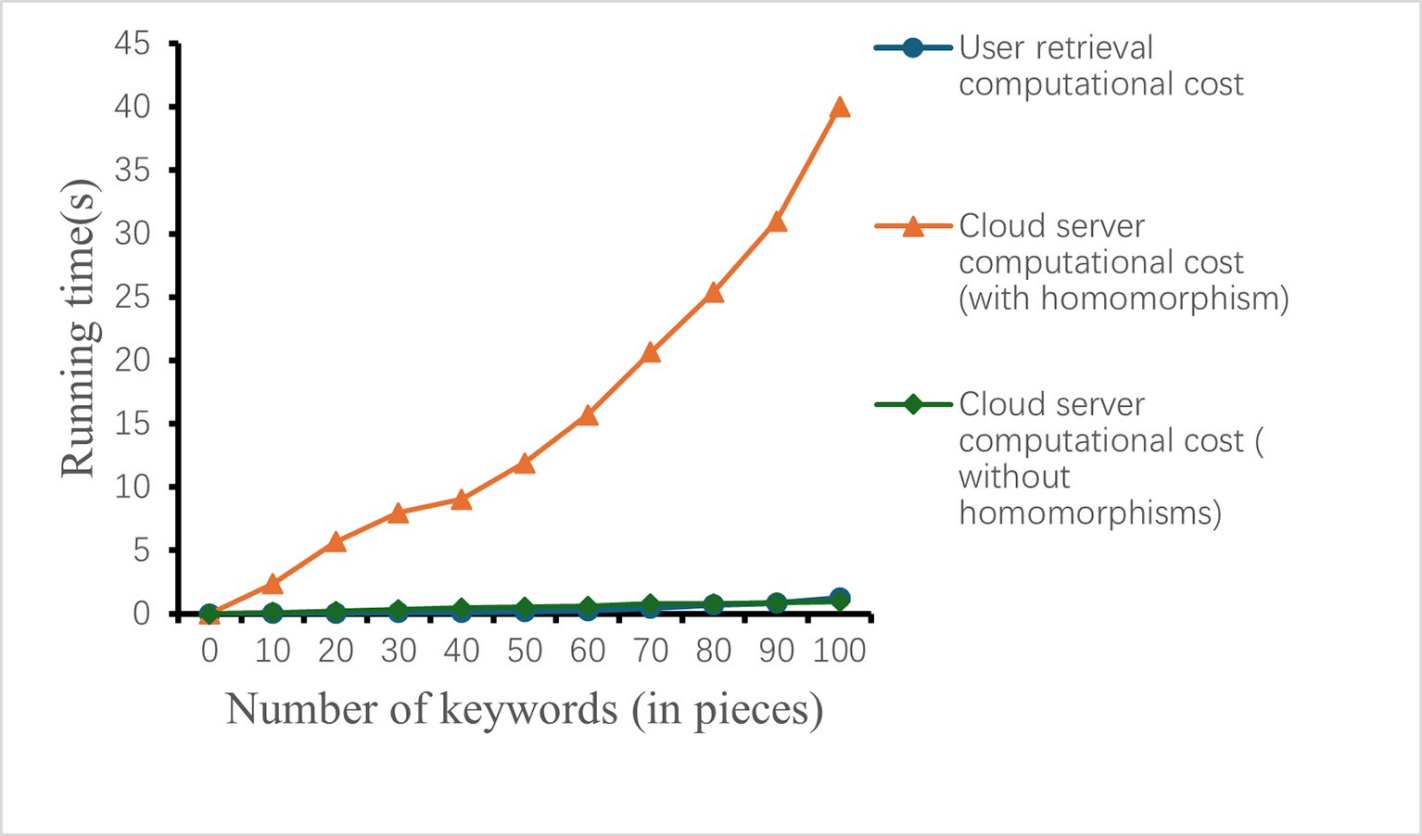
Computational costs with different process.

In Table 1, we give the calculation costs of user index construction cost, user retrieval calculating cost and cloud server computation cost, and we compare them with literatures [39–41] respectively. From Table 1, we can see that the proposed scheme cost is less than that of similar literatures. At the same time, the calculation cost of the entire retrieval process after using homomorphic encryption is also given in Fig 1, which shows that homomorphic encryption has a relatively large impact on the entire retrieval process. However, the calculation process of homomorphism is placed in the cloud computing server, and will not bring additional computing cost to the user’s retrieval process.

**Table 1:** Computational costs.

	User index construction cost	User retrieval calculation cost	Cloud server computation cost
[47]	2 *T*_*p*_ + *T*_*m*_	4*T*_p_ +*T*_m_	\
[48]	\	12 *T*_*r*_ +5 *T*_*m*_ +7*T*_*e*_ +4 *T*_*p*_	11*T*_*r*_ +(2*n*+4)*T*_*m*_+7*T*_*p*_+3*T*_*P*_
[49]	2*T*_m_+3*T*_*p*_	3*P*+(|*S*|−1)*T*_*m*_+3*T*_*m*_+ *T*_*e*_	2*P*+(*m*+3)*T*_*p*_+2*T*_*p*_+*T*_*m*_+(*m*+1)*T*_*m*_
Ours	2(*T*_c_+*T*_m_)+*T*_*a*_	2(*T*_m_+ *T*_*e*_+ *T*_*p*_+ *T*_*r*_)	3 *T*_m_+2(*n*+1)*T*_*p*_+3*T*_e_+*T*_c_

*T*_*c*_: Computational index cost; *T*_*a*_: Authentication cost; *T*_*e*_: Time consumption of exponentiation; *T*_*r*_: Generate random numbers; *T*_*m*_: Multiplication operation; |*S*|:Size of set S; *T*_*p*_: Pairing.

**Fig 2:**
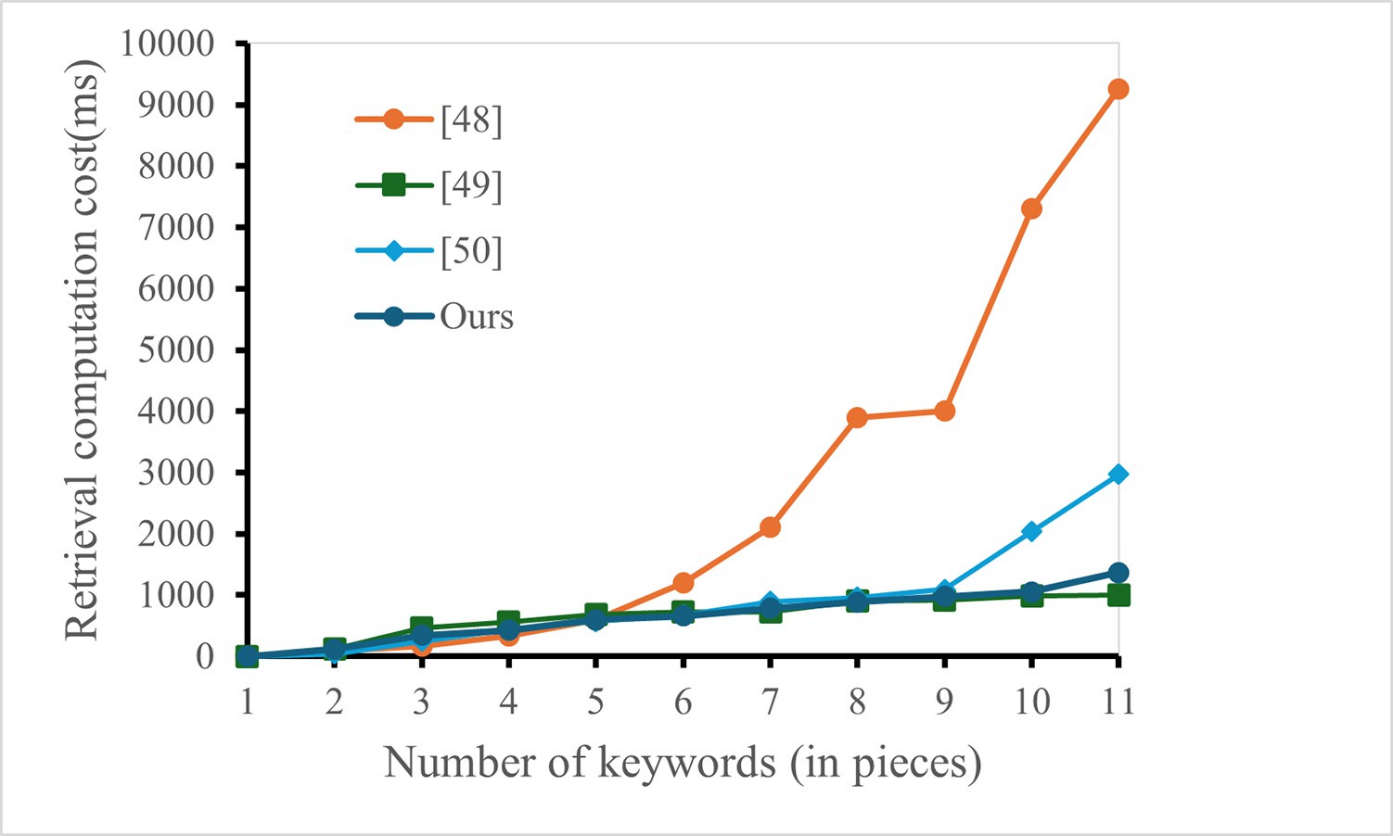
Retrieval computation cost.

**Fig 3:**
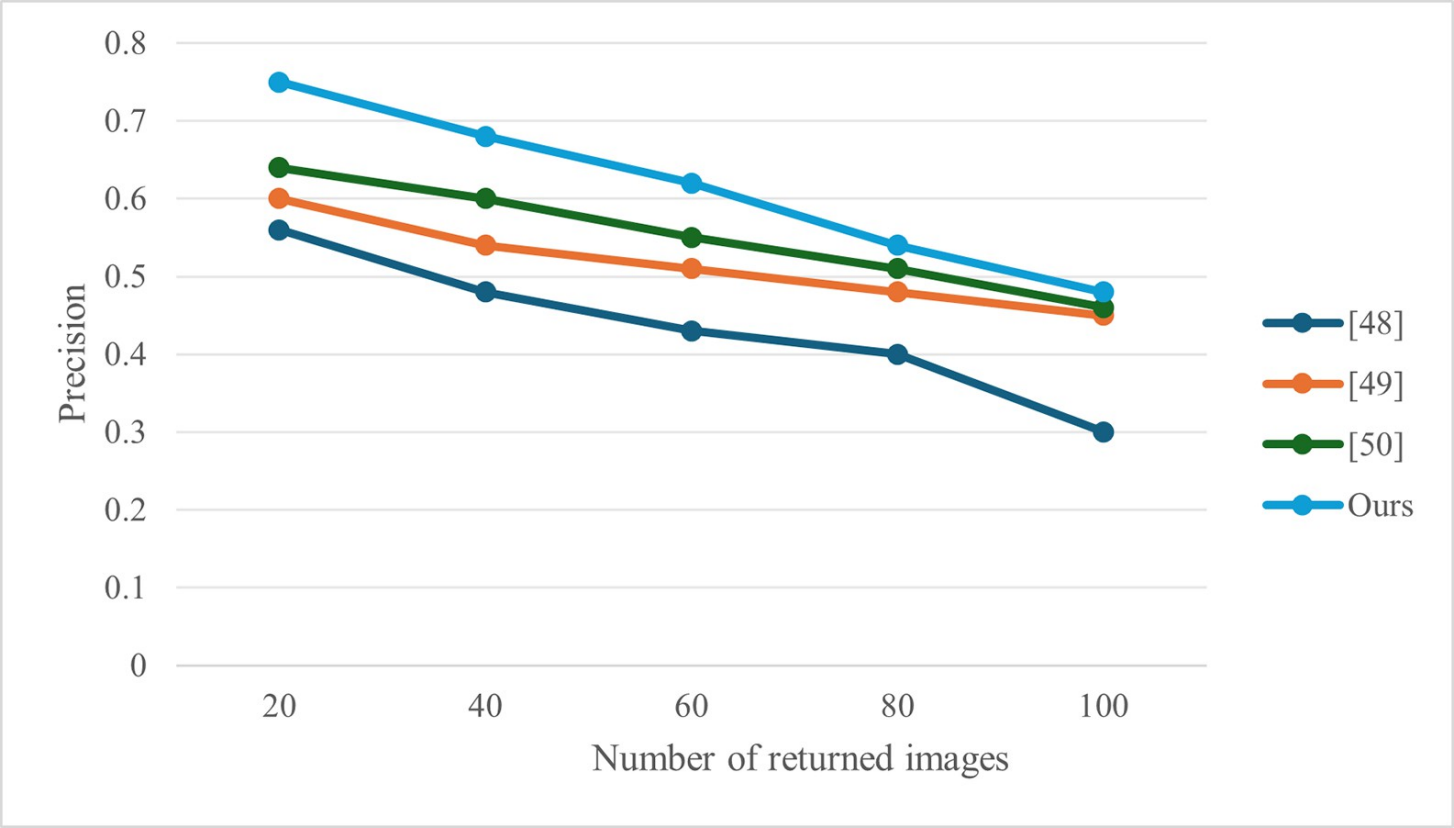
Retrieval accuracy.

The experimental analysis shows in Fig 2 that the computational overhead of the scheme proposed in terms of the computational overhead of user construction of indexes, the computational efficiency of the scheme in our scheme is better than the scheme proposed in [39, 41], and lower than the scheme proposed in [40]. But in general, the computational cost for retrieving users is not onerous. In addition, the scheme in this chapter is a dynamic authorizable ciphertext image retrieval scheme, which is more suitable for environments in the IoT where the data needs to be dynamically updated in real time. At the same time, in Fig 3, experiments were conducted to compare the accuracy and precision of retrieval with similar literatures. As can be seen from Fig 3, the retrieval accuracy of the scheme proposed in this paper is higher than that of similar literatures.

## V. Conclusion

In this paper, we offer a dynamic authorizable ciphertext image retrieval scheme based on security neural network inference that delivers considerable results in safeguarding user data privacy while also improving image retrieval security. We successfully generate efficient image indexes in an encrypted environment using secure neural networks for feature extraction, protecting the privacy of the source images. In addition, we present a dynamic authenticatable ciphertext retrieval mechanism that not only increases the system’s flexibility but also improves the users’ ability to obtain images within the approved range. Users can retrieve the required images quickly and accurately while maintaining data privacy, which further enhances the security of the system. In our experimental analysis, we comprehensively evaluate the user retrieval computation cost, user construction indexing cost, cloud computing computation cost (with homomorphism), and cloud computing computation cost (without homomorphism). And, while ensuring the user’s information security, it achieves a reasonable computational cost to fulfill the user’s needs in realistic applications. In summary, the research in this paper not only provides a new secure and flexible solution in the field of ciphertext image retrieval, but also serves a useful reference for the application of security neural network reasoning technology in other related fields. In the future, we will continue to explore and optimize the solution, in order to realize its full potential in more practical circumstances and provide consumers with more efficient and safe image retrieval services.

## Supporting information

S1 File Minimal data set. (XLSX)

## References

[1] Yang T, Ma J, Miao Y, et al. Mu-teir: Traceable encrypted image retrieval in the multi-user setting[J]. IEEE Transactions on Services Computing, 2022, 16(2): 1282–1295. doi: 10.1109/TSC.2022.3149962

[2] Hazra T K, Chowdhury S R, Chakraborty A K. Encrypted image retrieval system: A machine learning approach[C]//2016 IEEE 7th Annual Information Technology, Electronics and Mobile Communication Conference (IEMCON). IEEE, 2016: 1–6.)

[3] Anju J, Shreelekshmi R. Fsecbir: A faster secure content-based image retrieval for cloud[J]. Software Impacts, 2022, 11: 100224.

[4] Yang T, Liu Z, Guo J, et al. Image analysis by fractional-order weighted spherical Bessel-Fourier moments[J]. Pattern Recognition, 2024: 110872.

[5] Xia Z, Wang X, Sun X, et al. A secure and dynamic multi-keyword ranked search scheme over encrypted cloud data[J]. IEEE transactions on parallel and distributed systems, 2015, 27(2): 340–352.

[6] Jiang X, Yu J, Yan J, et al. Enabling efficient and verifiable multi-keyword ranked search over encrypted cloud data[J]. Information Sciences, 2017, 403: 22–41. doi: 10.1016/j.ins.2017.03.037

[7] Fu Z, Sun X, Liu Q, et al. Achieving efficient cloud search services: multi-keyword ranked search over encrypted cloud data supporting parallel computing[J]. IEICE Transactions on Communications, 2015, 98(1): 190–200.

[8] Cao N, Wang C, Li M, et al. Privacy-preserving multi-keyword ranked search over encrypted cloud data[J]. IEEE Transactions on parallel and distributed systems, 2013, 25(1): 222–233.

[9] Li H, Yang Y, Luan T H, et al. Enabling fine-grained multi-keyword search supporting classified sub-dictionaries over encrypted cloud data[J]. IEEE Transactions on Dependable and Secure Computing, 2015, 13(3): 312–325.

[10] Li J, Wang Q, Wang C, et al. Fuzzy keyword search over encrypted data in cloud computing[C]//2010 Proceedings IEEE INFOCOM. IEEE, 2010: 1–5.

[11] Liu C, Zhu L, Li L. Fuzzy keyword search on encrypted cloud storage data with small index[C]//2011 IEEE International Conference on Cloud Computing and Intelligence Systems. IEEE, 2011: 269–273.

[12] Wang J, Ma H, Tang Q, et al. Efficient verifiable fuzzy keyword search over encrypted data in cloud computing[J]. Computer science and information systems, 2013, 10(2): 667–684. doi: 10.2298/CSIS121104028W

[13] Shi J, Lai J, Li Y, et al. Authorized keyword search on encrypted data[C]//Computer Security-ESORICS 2014: 19th European Symposium on Research in Computer Security, Wroclaw, Poland, September 7–11, 2014. Proceedings, Part I 19. Springer International Publishing, 2014: 419–435.

[14] Yang Y, Liu X, Deng R H. Multi-user multi-keyword rank search over encrypted data in arbitrary language[J]. IEEE Transactions on Dependable and Secure Computing, 2017, 17(2): 320–334.

[15] Wang B, Song W, Lou W, et al. Inverted index based multi-keyword public-key searchable encryption with strong privacy guarantee[C]//2015 IEEE Conference on Computer Communications (INFOCOM). IEEE, 2015: 2092–2100.

[16] Guan Z, Liu X, Wu L, et al. Cross-lingual multi-keyword rank search with semantic extension over encrypted data[J]. Information Sciences, 2020, 514: 523–540.

[17] Sun W, Wang B, Cao N, et al. Privacy-preserving multi-keyword text search in the cloud supporting similarity-based ranking[C]//Proceedings of the 8th ACM SIGSAC symposium on Information, computer and communications security. 2013: 71–82.

[18] Song W, Wang B, Wang Q, et al. A privacy-preserved full-text retrieval algorithm over encrypted data for cloud storage applications[J]. Journal of Parallel and Distributed Computing, 2017, 99: 14–27.

[19] Zhong H, Li Z, Cui J, et al. Efficient dynamic multi-keyword fuzzy search over encrypted cloud data[J]. Journal of Network and Computer Applications, 2020, 149: 102469.

[20] Liu G, Yang G, Bai S, et al. FASE: A fast and accurate privacy-preserving multi-keyword top-k retrieval scheme over encrypted cloud data[J]. IEEE Transactions on Services Computing, 2020, 15(4): 1855–1867.

[21] Guo Z, Zhang H, Sun C, et al. Secure multi-keyword ranked search over encrypted cloud data for multiple data owners[J]. Journal of Systems and Software, 2018, 137: 380–395.

[22] Li X, Tong Q, Zhao J, et al. VRFMS: verifiable ranked fuzzy multi-keyword search over encrypted data[J]. IEEE Transactions on Services Computing, 2022, 16(1): 698–710.

[23] Chen J, He K, Deng L, et al. EliMFS: achieving efficient, leakage-resilient, and multi-keyword fuzzy search on encrypted cloud data[J]. IEEE Transactions on Services Computing, 2017, 13(6): 1072–1085.

[24] Al Badawi A, Bates J, Bergamaschi F, et al. Openfhe: Open-source fully homomorphic encryption library[C]//Proceedings of the 10th Workshop on Encrypted Computing & Applied Homomorphic Cryptography. 2022: 53–63.

[25] Bakshi M, Last M. Cryptornn-privacy-preserving recurrent neural networks using homomorphic encryption[C]//Cyber Security Cryptography and Machine Learning: Fourth International Symposium, CSCML 2020, Be’er Sheva, Israel, July 2–3, 2020, Proceedings 4. Springer International Publishing, 2020: 245–253.

[26] Cheon J H, Kim A, Kim M, et al. Homomorphic encryption for arithmetic of approximate numbers[C]//Advances in Cryptology–ASIACRYPT 2017: 23rd International Conference on the Theory and Applications of Cryptology and Information Security, Hong Kong, China, December 3–7, 2017, Proceedings, Part I 23. Springer International Publishing, 2017: 409–437.

[27] Chillotti I, Gama N, Georgieva M, et al. TFHE: fast fully homomorphic encryption over the torus[J]. Journal of Cryptology, 2020, 33(1): 34–91.

[28] Gentry C. Fully homomorphic encryption using ideal lattices[C]//Proceedings of the forty-first annual ACM symposium on Theory of computing. 2009: 169–178.

[29] Boemer F, Lao Y, Cammarota R, et al. nGraph-HE: a graph compiler for deep learning on homomorphically encrypted data[C]//Proceedings of the 16th ACM international conference on computing frontiers. 2019: 3–13.

[30] Arazzi M, Nicolazzo S, Nocera A. A fully privacy-preserving solution for anomaly detection in IoT using federated learning and homomorphic encryption[J]. Information Systems Frontiers, 2023: 1–24.

[31] Wang X, Garg S, Lin H, et al. Toward accurate anomaly detection in industrial internet of things using hierarchical federated learning[J]. IEEE Internet of Things Journal, 2021, 9(10): 7110–7119.

[32] Rey V, Sánchez P M S, Celdrán A H, et al. Federated learning for malware detection in IoT devices[J]. Computer Networks, 2022, 204: 108693.

[33] Rahmadika S, Astillo P V, Choudhary G, et al. Blockchain-based privacy preservation scheme for misbehavior detection in lightweight IoMT devices[J]. IEEE Journal of Biomedical and Health Informatics, 2022, 27(2): 710– 10.1109/JBHI.2022.3187037 35763469

[34] Meftah S, Tan B H M, Mun C F, et al. Doren: toward efficient deep convolutional neural networks with fully homomorphic encryption[J]. IEEE Transactions on Information Forensics and Security, 2021, 16: 3740–3752.

[35] Liu C, Zhu L, Chen J. Efficient searchable symmetric encryption for storing multiple source dynamic social data on cloud[J]. Journal of Network and Computer Applications, 2017, 86: 3–14.

[36] Miers I, Mohassel P. IO-DSSE: scaling dynamic searchable encryption to millions of indexes by improving locality[J]. Cryptology ePrint Archive, 2016.

[37] Chen Q, Kaleshi D, Fan Z, et al. Impact of smart metering data aggregation on distribution system state estimation[J]. IEEE Transactions on Industrial Informatics, 2016, 12(4): 1426–1437.

[38] Liu Y, Yu J, Fan J, et al. Achieving privacy-preserving DSSE for intelligent IoT healthcare system[J]. IEEE Transactions on Industrial Informatics, 2021, 18(3): 2010–2020.

[39] Stefanov E, Papamanthou C, Shi E. Practical dynamic searchable encryption with small leakage[J]. Cryptology ePrint Archive, 2013.

[40] Li J, Wang X, Gan Q, et al. MFPSE: Multi-user Forward Private Searchable Encryption with dynamic authorization in cloud computing[J]. Computer Communications, 2022, 191: 184–193.

[41] Li H, Yang Y, Dai Y, et al. Achieving secure and efficient dynamic searchable symmetric encryption over medical cloud data[J]. IEEE Transactions on Cloud Computing, 2017, 8(2): 484–494.

[42] Ahmed M R, Cano J M, Arboleya P, et al. Dsse in european-type networks using plc-based advanced metering infrastructure[J]. IEEE Transactions on Power Systems, 2022, 37(5): 3875–3888. doi: 10.1109/TPWRS.2022.3143695

[43] Zuo C, Sun S F, Liu J K, et al. Forward and backward private dynamic searchable symmetric encryption for conjunctive queries[J]. Cryptology ePrint Archive, 2020.

[44] Chen L, Li J, Li J. Towards forward and backward private dynamic searchable symmetric encryption supporting data deduplication and conjunctive queries[J]. IEEE Internet of Things Journal, 2023.

[45] Li Z, Ma J, Miao Y, et al. Forward and backward secure keyword search with flexible keyword shielding[J]. Information Sciences, 2021, 576: 507–521.

[46] Gilad-Bachrach R, Dowlin N, Laine K, et al. Cryptonets: Applying neural networks to encrypted data with high throughput and accuracy[C]//International conference on machine learning. PMLR, 2016: 201–210.

[47] Padhya Mukti, and Jinwala Devesh C. "MULKASE: a novel approach for key-aggregate searchable encryption for multi-owner data." Frontiers of Information Technology & Electronic Engineering 20.12 (2019): 1717–1748.

[48] Oh Jihyeon, et al. "A secure data sharing based on key aggregate searchable encryption in fog-enabled IoT environment." IEEE Transactions on Network Science and Engineering 9.6 (2022): 4468–4481.

[49] Liu Jinlu, et al. "Key-aggregate searchable encryption supporting conjunctive queries for flexible data sharing in the cloud." Information Sciences 645 (2023): 119336. doi: 10.1016/j.ins.2023.119336

